# Alpha-bisabolol, not a matter for cancer therapy. Commentary: “Research on the immunosuppressive activity of ingredients contained in sunscreens”

**DOI:** 10.3389/fphar.2015.00096

**Published:** 2015-05-11

**Authors:** Salvatore Chirumbolo

**Affiliations:** Department of Medicine, University Laboratories for Medical Research (LURM)-Medicine D, University of VeronaVerona, Italy

**Keywords:** cancer, bisabolol, therapeutics, apoptosis, leukemia, lymphocytes

A recent paper showed that bisabolol contained in cosmetics drastically dampened peripheral blood lymphocyte proliferation induced by phytohemoagglutinin (PHA) and enhanced the production of tumor growth factor-beta 1 (TGF-β1) on NCTC 2544 keratinocytes, although it did not change the activity of monocytes and dendritic cells (Frikeche et al., 2015). The authors showed that some organic molecules present in sunscreens impaired DC maturation, or inhibited lymphocyte proliferation as well as increased of TGF-β1 in the cell environment. Alpha-bisabolol [6-methyl-2-(4-methylcycloex-3-en-1-yl)hept-5-en-2-ol] is a sesquiterpene alcohol, present in different isomers (Figure 1) that has been described since many years as a promising anti-tumoral compound (da Silva et al., 2010; Seki et al., 2011) It reduces mammary tumor mass in mice and promotes the natural killer (NK) cells response (Costarelli et al., 2010). Alpha-bisabolol is present in *Matricaria chamomilla* L. essential oils and a potent pro-apoptotic molecule (Cavalieri et al., 2011). The myth of treating cancer with chamomile extracts would find unexpected support, as this plant contains flavonoids, as apigenin-7-O-glucoside and other phytochemicals, which act as anti-proliferative and pro-apoptotic molecules, (Srivastava and Gupta, 2007). Frikeche et al. showed that bisabolol behaves as a potent immuno-suppressant, an evidence that should raise fundamental issues about the role of plant-derived molecules on the tumor microenvironment, besides their direct effect on malignant cells (Frikeche et al., 2015).

**Figure 1:**
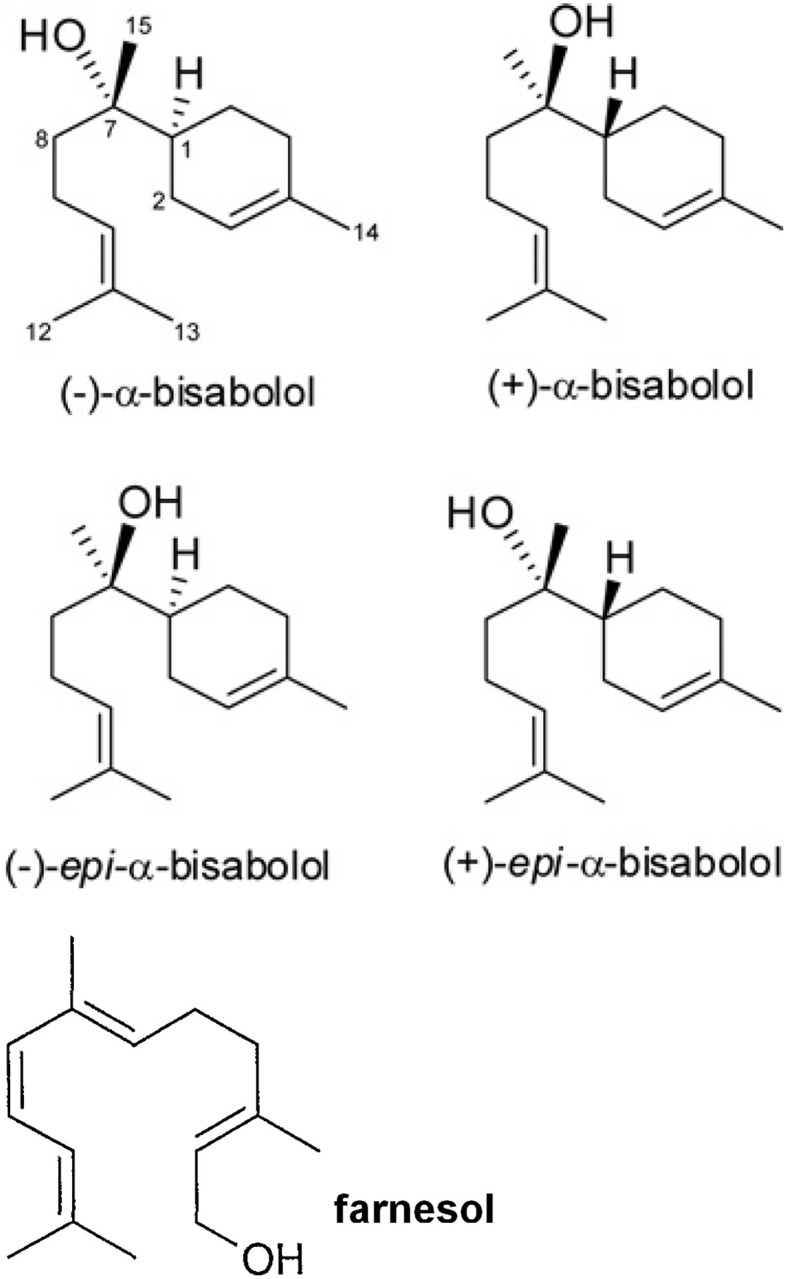
**Alpha-bisabolol different isomers and farnesol, a possible catabolyte**.

Darra et al., reported that the anti-neoplastic action exerted by α-bisabolol, derives fundamentally by its ability in inducing mitochondria-mediated apoptosis in cancer cells (Darra et al., 2007, 2008; Cavalieri et al., 2009). In particular, α-bisabolol is preferentially incorporated into malignant cells through lipid rafts and directly interacts with Bid protein (Darra et al., 2008). This mechanism, which may account for the reported anti-tumoral effect, has never been assessed *in vivo* and particularly Darra's *in vitro* evidence did not include the role of immune cells in the tumor microenvironment during α-bisabolol treatment. Promising results showed that α-bisabolol is active against primary acute leukemia cells, in synergism with tyrosine inhibitors, suggesting that its main target is the hematopoietic cell (Cavalieri et al., 2011; Bonifacio et al., 2012). Frikeche et al. would suggest that the immunosuppressive action performed by α-bisabolol on lymphocytes may have dramatic consequences on tumor development (Frikeche et al., 2015). Yet, some concern is about α-bisabolol and lipid rafts. Actually, gamma-delta phenotype T cells (TCR-γδ cells), increase lipid rafts when activated by involving membrane cholesterol (Kabouridis et al., 2000; Mahammad et al., 2010; Cheng et al., 2013). Due to its preferential entry through lipid rafts, α-bisabolol may induce apoptosis in activated T cells, while simultaneously switches off lymphocyte activation (Frikeche et al., 2015). Alpha-bisabolol tropism for immune cells may have fundamental effects on tumor immune microenvironment, probably by impairing T-cell activation and lymphocyte switching and promoting cancer editing, causing evasion from inflammation and generating immune tolerance (Vinay et al., 2015). Immune suppression in the tumor microenvironment is fundamentally mediated by CD4^+^CD25^+^FoxP3^+^ regulatory T cells (Tregs), as the major mechanism of tumor immune escape, a crucial hurdle for tumor immunotherapy (Jacobs et al., 2012). Bisabolol enhances TGF-β in *in vitro* cultured keratinocytes (Frikeche et al., 2015) and the cytokine is necessary for the progression of tumors such as hepatocellular carcinoma, acting by inducing Tregs polarization (Shen et al., 2015). In melanoma models, cancer cells induce immune escape and suppression by up-regulating CD4^+^CD25^+^FoxP3^+^ regulatory T cells, through TGF-β expression (Baumgartner et al., 2007). If α-bisabolol is able to increase TGF-β release, its chemopreventive potential might appear therefore quite controversial. At least apparently, α-bisabolol might induce immune suppression and tolerance by increasing the release of cytokines promoting cancer editing. Furthermore, α-bisabolol does not affect the ability of dendritic cells (DCs) to produce IL-12p70 (Johansson et al., 2011; Frikeche et al., 2015). DCs produce IL-12p70 after engulfment of apoptotic lymphocytes and this mechanism should induce immune tolerance in the absence of lymphocyte activation (Johansson et al., 2011). Furthermore, TCR-γδ cells are able to recognize several unknown antigens on tumor cells. Some metabolites of the mevalonate pathway, among which is farnesol, a possible catabolyte of α-bisabolol (Dewick, 2002), should act as tumor ligands, which can activate TCR-γδ cells (Gober et al., 2003). The role of TCR-γδ cells in tumors should appear encouraging (Hannani et al., 2012; Marquez-Medina et al., 2012), but these cells have also an immunosuppressive role when induced by TGF-β1 (Gu et al., 2014). Critical points to be addressed regards therefore the role of this sesquiterpene alcohol on immune regulation and hence on the immune competence in fighting cancer. This closely depends on the immune context where malignant cells are developing, besides to the bioavailability of α-bisabolol *in situ*.

Despite its promising activity as an anti-tumor molecule, α-bisabolol does not possess so different features respect to the widest family of plant-derived anti-inflammatory and chemopreventive polyphenols (Chirumbolo, 2010). The ability to induce cell apoptosis is shared with several other plant derived compounds, such as quercetin (Primikyri et al., 2014), genistein (Choi et al., 2007), apigenin (Papachristou et al., 2013), catechins (Yoon et al., 2014), resveratrol (Wang et al., 2011) and many others, for which these few examples are reported. The pro-apoptosis action should be interpreted at the light of the stress response mechanism activated by cells, a property shared by any plant-derived polyphenol, representing a general hallmark of these molecules (Fresco et al., 2010). Tumor cells have critically different patterns of stress response and they rapidly activate apoptosis pathway when stimulated by damage or stress signals, whose burden is particularly difficult to address. In this context a major role is played by endoplasmic reticulum stress (ER stress) and the unfolding protein response (UPR), besides to mitochondria (Maurel et al., 2015). While these mechanisms shed a light on the cellular impact of plant phytochemicals, their role on the cancer immune micro-environment is yet far to be fully understood. *In vitro* research usually neglected this issue, as most of investigations based on cell lines obviously never consider the immune microenvironment existing in the *in vivo* situation. In this perspective, the recent article by Frikeche et al., raises some criticism about the actual role of α-bisabolol as a real, promising chemopreventive molecule.

Alpha bisabolol might affect mitochondrial permeability transition also in non cancer cells (Leanza et al., 2013, 2014) and recent reports showed a massive death of endothelial cells by apoptosis induced from 5.0 μM α-bisabolol (Magnelli et al., 2010), a dose about 10-times lower than the one used to BCR-ABL cell viability in primary acute leukemia (Bonifacio et al., 2012).

As with other phytochemicals, the role of α-bisabolol on cancer therapy should be expanded in future debates, while any further proposals to investigate this organic compound on *in vitro* cancer lines, such as MiaPaCa, should be considered with caution.

## Conflict of interest statement

The author declares that the research was conducted in the absence of any commercial or financial relationships that could be construed as a potential conflict of interest.
